# Pupils’ Motivational and Emotional Responses to Pedagogies of Affect in Physical Education in Scottish Secondary Schools

**DOI:** 10.3390/ijerph18105183

**Published:** 2021-05-13

**Authors:** Cara A. Lamb, Eishin Teraoka, Kimberly L. Oliver, David Kirk

**Affiliations:** 1 School of Education, University of Strathclyde, Glasgow G4 0LT, UK; cara.lamb@strath.ac.uk; 2 Institute of Physical Education, Keio University, Yokohama 223-8521, Japan; eishin.teraoka@keio.jp; 3 Human Performance, Recreation and Dance, New Mexico State University, Las Cruces, NM 88003, USA; koliver@nmsu.edu; 4 School of Human Movement and Nutrition Sciences, University of Queensland, Brisbane, QLD 4072, Australia

**Keywords:** physical education, pupil motivation, pupil voice, pedagogy of affect, activist approach

## Abstract

This paper reports on the findings of two studies concerned with pupils’ motivational and emotional responses to pedagogies of affect in physical education in Scottish secondary schools. Pedagogies of affect explicitly focus on learning in the affective domain, or what is known in Scotland’s Curriculum for Excellence (CfE) as ‘personal qualities’. Personal qualities include motivation, confidence and self-esteem, determination and resilience, responsibility and leadership, respect and tolerance, and communication. In one study, led by Teraoka, the researchers explored the ways in which pupils responded, through focus group interviews based on Self-Determination Theory, to teachers who claimed to value and be committed to teaching explicitly for affective learning outcomes. In another study, led by Lamb, the researchers investigated the impact of an activist intervention on girls’ experiences of physical education, through their conversations in focus group discussions. Both studies reveal that pupils responded favorably, both in motivation and emotion, to pedagogies of affect in physical education, and that these responses offer a promising basis for future developments.

## 1. Introduction

In terms of their contribution to the health and wellbeing of children and young people (CYP), since at least the 1980s, physical educators around the world have been exhorted to attend to obesity as the major public health issue of the day (e.g., References [1,2,3]). Advocacy for particular kinds of physical education programs in schools has followed. The incorporation of Moderate to Vigorous Physical Activity (MVPA) into school programs has been viewed as one essential requisite of these physical education programs. Despite the widespread adoption of initiatives, such as Fitnessgram^®^, in the USA [4], and the increasing visibility of physical activity and physical fitness concepts in national curricular policy in a range of countries [5] the physical education practitioner community has been somewhat ambivalent about the obesity focus of their contribution to CYP’s health and wellbeing (e.g., Reference [6]).

We can only speculate on the reasons for this ambivalence. Perhaps, as Kirk [7] argues, the traditional and dominant multi-activity form of physical education in schools based on sports techniques has kept concerns for health-related physical activity on the subject’s margins, even though fitness testing continues to thrive, inappropriately [8], within such programs. Perhaps, also, physical education teachers understand that their programs offer educational benefits to CYP that go beyond MVPA and physical fitness. Indeed, physical educators have traditionally viewed learning in the affective domain as an important health-related outcome of their teaching. At the same time, within this traditional perspective, these outcomes are mainly viewed as hoped-for byproducts of physical education as opposed to intentional curricular foci.

Recently, however, there has been increased attention to ‘pedagogies of affect’ among the scholarly community, where learning in the affective domain is planned-for and intended rather than merely hoped-for [9]. Kirk [9] proposes that well-known and long-running forms of physical education, such as Teaching Personal and Social Responsibility (TPSR) [10], activist approaches to working with girls [11], and adaptations to sport education, such as Sport for Peace [12], can be viewed as exemplary pedagogies of affect. Moreover, research on pupil motivation in physical education contexts, much of it informed by Self-Determination Theory (SDT), has also brought the idea of teaching for affective learning to prominence in the pedagogy research literature [13].

As curriculum policies in various nations and regions around the world have been shaped over the past decade and a half by a notion of physical education-as-health promotion [5], learning in the affective domain has also been prominent. In Scotland’s Curriculum for Excellence (CfE), for example, affective learning, characterized as ‘Personal Qualities’, is included as one of four benchmarks for learning, the others being Physical Competencies, Physical Fitness, and Cognitive Skills. Personal Qualities include learning aspirations for motivation, confidence and self-esteem, determination and resilience, responsibility and leadership, respect and tolerance, and communication.

We think this turn towards learning in the affective domain as a health-related concern of physical educators is timely and important. The OECD [14] observed that mental illness is the largest category of disease among CYP. They also state that around half of all mental health problems begin by the age of 14 and three-quarters by the mid-20s. In a review of reports on the mental health of 4–25-year-olds, over a 19-year period between 1995 and 2014 in Britain, Pitchforth et al. [15] found a six-fold increase in the prevalence of mental illness in England, and more than double between 2003–2014 in Scotland. They describe these increases in prevalence as ‘striking’. We should note that these figures predate the COVID 19 pandemic. In an update on the impact of the pandemic, the Children and Young People’s Mental Health Coalition (CYPMHC) [16] in Britain reported that 83% of CYP with pre-existing mental health problems said that their problems had worsened. The closure of schools during the several lockdown periods of the pandemic in Britain meant that there was limited capacity for teachers and health workers to recognize which CYP were experiencing mental health problems, with restricted access to support services generally [16].

This emerging crisis in the mental health of CYP requires us to rethink the dominant view that physical education’s main contribution to their health and wellbeing is through programs that promote MVPA and physical fitness. If obesity is not the only health problem affecting CYP, then we require additional pedagogical strategies to MVPA in physical education. The purpose of this paper is to report the findings of two projects carried out in secondary schools in Scotland, which focused on pedagogies of affect. A study led by Teraoka, he and Kirk explored the ways in which pupils responded to teachers who claimed to value and be committed to teaching explicitly for learning outcomes within the Personal Qualities aspect of physical education in the CfE. A second study led by Lamb, she, Oliver, and Kirk investigated the impact of an activist intervention on girls’ experiences of physical education through their conversations in focus group discussions. Our findings suggest that pupils recognized and approved of their teachers’ intentions to teach for affective learning, and that they were benefitting from this approach to physical education.

Our research objective in this paper is to report on pupils’ motivational and emotional responses to pedagogies of affect in secondary schools in Scotland. In the next section of the paper, we outline the research designs of each study briefly. Then, we report the findings of each study, and conclude with a discussion of the issues raised by both in terms of pupils’ motivational and emotional responses to pedagogies of affect.

## 2. Research Designs

State-funded comprehensive schools cater to approximately 96% of school-age children in Scotland. State sector schools were the setting for these studies. Study 1 was conducted in three non-denominational schools, whilst Study 2 took place in two Roman Catholic schools. School A in Study 2 was an all-girls setting. Schools and teachers were recruited using purposive sampling [17].

### 2.1. Study 1

A mixed-method approach was adopted for this study, though only qualitative data is reported here. We used professional contacts to recruit a purposive sample of teachers who had an expressed commitment to teaching explicitly for Personal Qualities. Qualitative data were generated from pupil focus group interviews with pupils aged 11–14 years. A total of 32 pupils from four different classes in three schools were involved in the interviews reported here. The teachers in School a were ‘Lisa’ and ‘Steven’, in School b ‘Chloe’, and in School c: ‘Luke’. All the teachers’ names are pseudonyms. The data collection of this study ran from October 2018 to May 2019.

In terms of data generation, Teraoka observed two indoor lessons for each class. Within a few weeks after the lessons, selected pupils participated in focus group interviews. Each focus group interview was completed within approximately 30 min. We asked pupils questions about how their teacher helps them to be motivated and engaged in physical education.

Qualitative data were transcribed and identified themes in relation to pupils’ perceptions of teaching behavior grounded in SDT.

### 2.2. Study 2

The data from this study were generated over a period of nine months (from August 2016 until April 2017). During this period, two teachers (‘Jess’ and ‘Kate’) engaged in an intervention using an activist approach [11] when teaching their year 4 secondary core^1^ physical education class. Jess taught in School A and used this approach for two of her classes. Kate taught in School B and used this approach for one of her classes. Both classes were single-sex, and the girls were all in the same year group (age 15–16 years old). Both teachers were recruited for a pilot study (see [18]) prior to this year. During the pilot, they were introduced to the activist pedagogical model. The data from this study was generated during what Goodyear and Casey [19] refer to as the ‘honeymoon period’ of pedagogical innovation.

The Study 2 data presented in this paper is from focus group interviews with the girls. Around 30 girls from three different classes were involved in these discussions that occurred twice throughout the study, once in November and then again in March. Further data were generated from teacher interviews, lesson observation field notes, and Skype video conversations with Oliver, the expert in the activist approach who introduced this to Lamb and the teachers. Although most of these data are not reported here, they helped triangulate the analysis and authenticate the activist experience in the field. For this paper, we considered that foregrounding the girls’ experiences, and in turn, their voices, would best exemplify the girls’ motivational and emotional responses to an activist approach as a specific pedagogy of affect.

## 3. Results

### 3.1. Study 1

We will report pupils’ responses to teaching behavior as predictors of pupils’ engagement, motivation, and psychological wellbeing under two themes (1) social support from teachers; (2) clarifying expectations. The context of the class setting will be featured as a significant class environment for implementing pedagogies of affect.

#### 3.1.1. Social Support from Teachers

There was evidence to show that the female teachers were successful in enhancing girls’ motivation and determination in girls-only classes. Lisa taught a basketball lesson for S1 pupils in a girls-only class. In the focus group interview, the girls commented on how Lisa helped them to be motivated and engaged:

Pupil 1: She motivates you and just encourages you and she make you be determined.

Pupil 2: She doesn’t force you.

(S1 Girls Focus Group, School a)

Likewise, the pupils’ perceptions of Chloe’s teaching behavior were similar to Lisa’s class. Chloe taught a lesson to a girls-only class. Girls commented:

Pupil 1: She’s really encouraging. She never makes people feel bad about themselves. She tries to help people be the best that they can.

Pupil 2: If there’s something that you can’t do, she doesn’t make you keep trying. She wants you to be able to do it, but she doesn’t keep making you try it. She just tells you to take tries and that’s going to be easier than build up to it.

(S2 Girls Focus Group, School b)

These girls do not want teachers to force them to do something if they were not able to do it. This kind of interaction made girls feel comfortable with teachers. This notion was also related to the relationship with teachers. In another focus group, a girl recognized that the relationship with their teacher is a factor in engaging in lessons.

You can trust your teacher, ‘cause if you don’t have a very good relationship, then you’re not going to trust them. If they tell you how to do something and you don’t trust them, you’re not going to believe them.

(S2 Girl, School b)

According to SDT, these girls seemed to have adequate relatedness need satisfaction to their teacher, which could contribute to their motivation and engagement.

With regard to pupils’ perceptions of the relationship with their teacher, there was a case of a coeducational class, taught by a male teacher, Luke. In a focus group interview with boys, there were different notions about the relationship with the teacher. On the one hand, a boy did not care about his teacher, as exemplified by the following comment:

I don’t necessarily connect with him. He just sees in school.

(S3 boy, School c)

On the other hand, another boy commented on his perception of the teacher in general:

He’s always given me a second chance. Like if there’s been doing wrong sometimes, he’s always been fine with me the next day. He’s always given me another chance which I respect.

(S3 boy, School c)

In another focus group of girls from Luke’s class, a girl commented that they feel the teacher knows them well.

They get to know you individually rather than as a class. If something happens in the class, they don’t associate it with the full class. They’ll get to know what’s happened.

(S3 girl, School c)

The results showed that pupils’ perceptions of teachers were considerably different from each other because teachers might interact differently with different pupils. It might be necessary to focus on individual teacher-pupil interactions, particularly teachers’ interaction with a pupil who had a negative perception.

#### 3.1.2. Clarifying Expectations

Pupils had a positive emotional response when they perceived that teachers clarify expectations for learning outcomes. For example, a pupil from Lisa’s class commented on teaching behavior concerning the provision of structure within SDT, which was helpful to clarify the contents of learning.

Before we start, there’s the board, and she always shows us what to do. Then after we discuss how to improve on what we’ve done.

(S1 girl, School a)

The interviewer recognized that the board was in the gym. It illustrates the four benchmarks in physical education, and teachers wrote out a target for each lesson and success criteria. As the girl commented, it seemed helpful for teachers and pupils to ensure what today’s focus is and what was to be learned today.

There was another case of male teacher, Steven, who taught a badminton lesson for S3 boys and girls in a coeducational class. In a focus group interview, boys highlighted that Steven was helpful to engage in lessons because the teacher provided explicit instruction, which refers to the provision of structure. For example, a boy commented:

If you’re doing something wrong, he’ll come and tell what you’re doing wrong and show you how to do it properly. And that helps.

(S3 boy, School a)

Additionally, another boy commented that Steven made him feel positive when he was distracted:

Sometimes I’ll misbehave a bit or be distracted easily so he’ll say this to me stuff like “I know you can do better this”, and that makes me feel better because that makes me think what I can actually do.

(S3 boy, School a)

This result seemed to show that the teacher brought positive outcomes for some pupils who need direct teaching, although the pupils’ perceptions of control teaching were high. Still, teachers might need to adopt different teaching styles according to individuals’ needs, preferences, and interests.

### 3.2. Study 2

The girls from the three-core physical education (PE) classes had different emotional and motivational responses to the activist intervention, both between schools, classes, and between the girls themselves. The data from the girls also revealed a contrast between the girls’ experiences of the activist program and the more traditional programs they experienced prior to this study. Two emerging themes arose from the analysis: (1) The motivating and de-motivating practices in physical education, and (2) the change in power dynamics within the classes.

#### 3.2.1. The Motivating and De-Motivating Practices in Physical Education

In conversation with the girls, they were able to articulate the positive experiences that they had when their teachers used the activist pedagogy during their core PE classes. In particular, they found that choice, novelty, and variety were motivating practices that facilitated their engagement in PE. They also reported on how the inclusive practices their teacher offered during the lessons helped them feel more comfortable and gave them more confidence, which led to an increase in participation. In contrast to motivating practices, the data found that when choice, novelty, and variety were not part of PE programs, the girls were not motivated to participate. Furthermore, when the girls did not feel comfortable and included in their PE lessons, they were less willing to participate.

##### ‘*Everyone Enjoys it More’*—Choice, Novelty, and Variety

As the girls were experiencing learning in a ‘new’ way (i.e., through an activist approach), they found more enjoyment in their PE lessons. One reason was that they were offered choices during the sampling lessons that were a feature of the Building the Foundation (Btf) phase of the approach (see [20] for an overview of this). When asked about what was different about PE, choice was given as a common factor in the girls’ enjoyment of PE. During the focus group interviews with School B, one girl commented that ‘everyone enjoys it more (…) because we’re doing the things we like’. Another pupil added that she ‘used to kind of dread PE’, but she enjoys it more now ‘because (the class has) picked (the activities)’. These strong negative emotions of ‘dreading PE’ tell us something about how the girls experienced PE previously. It may be that their previous and more traditional teacher-directed PE programs provided less choice to the girls with the teachers making all of the decisions, and this could be a de-motivating factor in adolescent girls’ engagement. This was emphasized when one girl added that they ‘just got [sic] made to go outside whether it was freezing (or not…) and (their teachers) would stand with their big jackets on.’ Not only does this tell us about the lack of choice offered, but it also gives some insight into the expectations of their previous teachers, i.e., forcing pupils to participate in PE in cold weather whilst teachers stood at the side with their warm clothing on.

Girls from School A also commented about how lack of choice was a hindering factor in their motivation during previous PE lessons with other teachers. This short discussion below highlighted similar issues the girls from School B mentioned above. In their previous programs, they did not have the opportunity to choose any physical activities they participated in, and their teachers made all the decisions. In fact, Pupil 1 conveyed her negative emotions about her previous PE program by stating that she ‘hated it’.

Pupil 1: My old teachers were on the whole strict, so if you didn’t bring in PE kit you got detention. And you didn’t get to choose, like you had something to do and you had to do it.

Pupil 2: Did you like not getting to pick?

Pupil 1: No I hated it because you had to (…) do gymnastics and I’m terrible at it.

Another aspect of choice that the girls from both schools responded positively to was how their teachers offered them a variety of modifications within lessons in terms of the level they felt comfortable working at. Examples of these choices within lessons were when the teachers offered the girls an alternative exercise or when they didn’t push them too hard (referring to exercise). One girl from School B explained how Kate, her teacher, gave her ‘an easy option, (…) a hard option and a middle option’ when she participated in a legs, bums and tums lesson. Another girl commented how Kate allowed the girls to work ‘at (their) (…) own pace’. Both girls agreed that the offering of choice within the lesson motivated them to try harder. Although we understand that choice in PE programs has been reported as a positive influence for engagement in PE (see [21,22,23]), embedding choice throughout full programs and lessons was afforded to teachers, Jess and Kate, when they implemented the activist approach.

Having control over choice had a direct impact on the girls’ self-determination. However, this was not the only motivating factor in the girls’ PE lessons. They also responded positively to the variety and novelty of the activities they were taught. During the BtF phase of the activist approach, teachers sought to broaden pupils’ perspectives of what is possible in PE. To do this, teachers co-created their ‘taster program’ with the girls’ interests central to what activities they were offered. As part of the taster program, girls were taught a variety of activities that were non-traditional. By non-traditional, we mean physical activities that were not part of their regular PE programs. Examples of some of the activities offered by the teachers were yoga, trampolining, cardio-games, Zumba, punchball, legs, bums and tums, etc. The purpose of this was to give the girls opportunities to experience different forms of physical activity that they might want to pursue more in the future.

Girls from both schools responded positively to the novelty and variety the activist program catered for. Thus, they were more engaged in their PE lessons. One girl from School B said that she ‘enjoy(ed) it more because (she was) not going to come in (to PE) and do the same thing over and over again’. Another girl found that when the teacher revealed the new activity to the class, she was ‘a bit more excited for it’. Other girls from School B were able to contrast their experience of a more traditional program with the activist program. They found they ‘everyone in the class took part’ when they were ‘doing (…) different things’. During the focus group discussion, girls provided an example of how a novel activity, such as Zumba was something they looked forward to:

Pupil 1: Like I would never do Zumba (outside of school) but then I tried it in school.

Interviewer: Okay so you’re trying new things now that you wouldn’t have done before?

Pupil 1: Yeah

Pupil 2: Like it gets you excited (…) for going to the class as well, like you look forward to it.

(Pupil Focus Group, School B)

We can interpret from these discussions about choice, novelty, and variety that the pupils from this study were more engaged in their lessons when these factors were part of their PE programs. Moreover, as teachers adopted an activist approach, in which the girls’ interests and motivations to participate in physical activity were central, the teachers were able to codesign their program with the girls. By intentionally listening and responding, a critical element of an activist approach, the teachers learned that to better facilitate student interest, motivation, and learning, they needed to consistently embed choice within their lessons, and that novelty and variety were not only enjoyable, but necessary. This is consistent with other findings from activist studies [20,24]). Additionally, as the girls from the focus group were so vocal about these factors being part of their PE experience during the fieldwork, we might assume that they are not regular features in the more traditional form of PE that they had normally experienced, i.e., the multi-activity short block program. This suggests that the multi-activity approach often lacks choice and teachers tell pupils what they are doing; it has less variety, and the same activity is repeated for 6–8 weeks; and it is often designed around the same sports/activities year on year, lacking in more novel activities.

##### ‘*People Don’t Judge as Much*’—Inclusive Practice and Feeling Comfortable in the Environment

Another feature of the activist pedagogy that the girls experienced was how inclusive it was. The girls in both schools revealed that when they were more comfortable in the environment, everyone was more involved. Furthermore, they felt more confident in themselves when the learning environment was comfortable, which in turn led to an increase in engagement. In contrast, if they were put into positions where they felt less comfortable (i.e., when they were forced to do something or when they felt judged), their levels of participation decreased. We know from previous activist studies with girls (for example, [23,25,26,27]) that when the researchers centralized the girls’ embodied experiences pedagogically, they were able to support the girls they worked with to experience a more inclusive PE environment where they felt more comfortable and more confident.

Feeling comfortable in the PE environment was central to the girls’ engagement in this study. Girls from School A commented that they felt comfortable when their teacher, Jess, didn’t push them too far. One girl commented how her teacher didn’t force her to wear bibs as she didn’t like wearing them. Another girl added that ‘if (the class didn’t) feel comfortable with something, (…) she (Jess) doesn’t push (them). [Adding] Like she pushes us but not so far so that we are feeling uncomfortable.’ We can interpret from this that being forced to do something in PE can lead to a negative experience. Indeed, this negative experience of not feeling comfortable may be a feature of programs where girls embodied experiences are not central to their PE program. This could suggest that more traditional programs rarely place pupils’ embodied experience (or their feelings of comfort) as a design feature of physical education curricula.

The embodied experience of feeling comfortable was echoed by the girls in School B when they spoke about how being in an all-girls class helped them feel less judged. They spoke about how ‘people don’t judge as much’ and how they ‘don’t feel like there’s any pressure as nobody’s judging you’. Particularly, these girls were clear about the effect of the presence of boys had on their involvement in PE lessons:

Interview: And do you think if boys were there, you’d feel judged?

Pupil: Yeah like see like with boys in my (other) PE class, when they do basketball, they’re like so rough, so we just tend to like stand on the sides and don’t do much.

Interview: So, you don’t get involved? And do you get involved in this class more?

Pupil: Yeah

The presence of boys and the ‘rough’ way that they played led to some girls become less involved in PE as they would ‘stand on the sides’ not doing much. It’s important to note that the activist approach was not implemented in the ‘other’ PE class mentioned above. However, importantly, this girl understood that there was a difference in her level of involvement in the two classes. In her ‘activist’ class, her peers did not judge and were not ‘rough’ like the boys in her ‘other’ class were.

A critical element of the activist approach involves a teachers’ attentiveness to issues of embodiment. Part of this requires that teachers work with their female students to co-create an environment where girls can feel comfortable. This study found that Kate, the teacher from School B, made some conscious actions to ensure no one was left out and that the girls felt comfortable. One pupil described this in the focus group discussion:

Like there’s some other PE classes where people can just go unnoticed and not get spoken to the whole class but (…) Miss (Kate) will (…) make an effort to speak to everyone and make sure they feel involved.

She added:

Like if we’re (…) picking teams (…) everyone will always have a certain group they’ll go with and then sometimes there will be a few people that will be left. Or if (…) there’s a group and there’s one space left, they’ll get split up and put there. Like she (Kate) always makes sure that everyone’s with (…) one or two other people they want to go with and then she’ll put two groups together so you’re always with someone that you want.

And when asked how this makes them feel, one girl said:

It’s better cos (…) see when there’s (…) people who are quite shy and you might not speak to them, you’ve already got a friend there and it’s easier to speak to people when it’s already two.

From the comments above, we can see how Kate made an intentional effort to help the girls get to know each other. By ensuring that no one was left out when making groups, Kate made sure the girls felt comfortable, and because of this, more people participated. The teachers’ use of an activist approach in PE allowed the girls to experience a much more inclusive environment, one that focused specifically on their needs as opposed to their teachers’ assumptions of their needs. We understand from these examples of inclusive practice that the girls were motivated to participate and no longer experienced a ‘dreaded feeling’ when going to their PE lessons. They valued having their voices both sought, taken seriously, and responded to pedagogically. They appreciated their teachers because their teachers showed appreciation for them. While seemingly simple, it is the honoring of pupil’s voice that facilitates pupil engagement, which ultimately might lead to lifelong physical activity participation.

#### 3.2.2. The Change in Power Dynamics

When Kate and Jess chose to work with these S4 classes using an activist approach, something they noticed straight away was the ‘split’ in their classes, divisions between groups of girls who didn’t know each other and so did not interact comfortably. As activist teachers, they knew that it was important for them to start where the girls are [11] and moved forward from there. Jess noticed this split when the girls were vocal about how their peers didn’t always listen to each other during a class discussion. Kate also recognized this when putting the girls into groups, and they didn’t really speak to each other. These reflections from the teachers became something that they knew they had to work on during their lessons if they wanted the girls to become more motivated in PE. The pupils were able to recognize that their teacher’s intention to help them get to know one another led to an improvement in their PE experiences.

##### ‘*I Genuinely Feel Like We’re a Family*’—A Sense of Togetherness Develops Amongst Pupils

As discussed in the previous section, cocreation of an environment where pupils feel comfortable and listened to is key to activist work. When implementing the activist approach in their own context, both teachers took time to help girls in their classes get to know each other. One girl spoke about how Jess ‘splits everyone up and puts them in random groups’. This led her to ‘feel more confident talking in PE’. Additionally, Girls from Kate’s school (School B) discussed some of the activities she did with them to build relationships:

Pupil 1: I think it’s because (this year) we started off where (…) we discussed like what we wanted to do and I think she’s also like took that and like she’s considered what we wanted to do in PE (…) and we’ve also discussed how to act around others and how to respect them and that.

Interviewer: Okay tell me about that.

Pupil 2: We did a like a get to know thing and we got to know people better in the class.

Pupil 3: Like team building things.

This explanation demonstrated how Kate sought pupils’ voices initially when co-creating an environment of respect. Another girl from School A explained a team building activity that Jess did with them to develop trust amongst pupils:

We did an activity where there were benches and everyone had to stand on a bench and one person at the top had to get through everyone on the bench to get to the bottom and we had to kind of trust each other to get the person to the end.

What is important to understand here is that ‘normally’ time is not given over in PE programs to develop these relationships between peers. However, as the teachers were implementing an activist approach, they devoted time to co-creating an inclusive environment, and this was central for them to work together as a group. The initial lessons in the BtF phase identified a ‘split’ in each of the classes, and therefore, the teachers took immediate action to rectify this issue, which is evident from the activity descriptions from the girls in the focus groups. As the ‘split’ in the classes became less visible, a sense of togetherness developed between the girls. Girls from School A commented on this:

There’s this other girl who used to be quite shy when it comes to PE and she used to be quite anxious and nervous whenever we’d be told we’d be doing new things especially if it involved having to work really hard and maybe not achieving as much as the people around you, she’d get really quite worried. But, I think as a class if we ever saw her upset we’d help her out and be like oh you don’t have to worry we’ll do this together.

(Pupil 1)

I feel like we all have changed. Like don’t judge me but I feel like we are like a little family, like I genuinely feel like we’re a family.

(Pupil 2)

This feeling of connectedness can be related to how confident the girls felt around each other and had a direct effect on their engagement in their PE lessons. In the first comment, the girl spoke about how the girls in her class would help a girl who was ‘anxious and nervous’. This seems like the girls were standing up for their peers, who did not always feel confident in standing up for themselves. In fact, they seemed to all be in ‘this together’, ‘like a little family’. Recognizing less confident peers and feeling as comfortable as you do with family was a huge factor in co-creating an atmosphere in PE where the girls felt more like equals, and this shifted the power relationships in their class.

##### ‘*Nobody Is Better Than Anyone Else’*—The Shift in Power between Peers

Stemming from a sense of togetherness emerging with the PE classes, the data also revealed that the girls felt strongly about the shift in the power between peers in the class. Girls from School A spoke about the louder, more dominant girls. They felt that since beginning their activist program, ‘the loud people’ ‘don’t control’. These more dominant girls were often seen as those who would ‘boss everyone about’, but now ‘they talk to everyone’. One girl summed this up nicely when she commented on how she felt the environment in PE was like now:

I feel like it’s that way when we come into PE, we all kind of just, we’ve all become the same level, like nobody is like better than anyone else.

Articulating that there were no longer girls in the class that was better than anyone else suggests that the power relations between peers had shifted in the class and the dominant girls were no longer ‘(bossing) everyone about.’ Another girl from School A added to this:

There’s also the attitude of working with others. Like some people may be used to get really angry and were very loud about certain things, like if they felt (…) their opinion maybe wasn’t being heard… But now (…) they’ve got calmer and they actually (…) cooperate.

This suggests that the dominant girls were now calmer (or no longer ‘loud people’), and that the girls were now cooperating more with each other. Additionally, this helps us understand more about what the girls had experienced prior to the activist intervention. Before this intervention, we can gather that they experienced a PE class where the dominant girls took over; there were people who were better than others; the less confident voices were not heard, and pupils did not always cooperate with each other. Although, we would never suggest a traditional program would consist of these features, we can determine that the activist approach was purposeful in its methods to challenge this. The data from the girls provides evidence that the co-creation of the environment between teachers and pupils was central to shifting the power dimensions between peers in the class.

##### ‘*If She’s Doing it, She Can Like Understand’*: Teachers’ Democratization of Their Practice

Another way to disrupt power dimensions with the class was through the teachers’ democratization of their practice. In this sense, teachers shift to a more student-centered pedagogy in which they ensure that their practice is ‘accessible to all’. There was evidence to suggest that both Kate and Jess were able to challenge a ‘one-size fits all’ approach of traditional practice and democratize their practice within their core PE classes.

One way that Kate did this was by joining in with her class during her PE lessons. One girl from Kate’s class commented that she liked this because if ‘(the teacher is) doing it she can understand… the pace’. This girl also said that her other teachers before ‘would just stand’ during the lesson. Later in this focus group discussion, the girls displayed emotions when they discussed this practice of teachers ‘shouting’ and ‘standing’:

Pupil 1: Because if different (…) teachers are just shouting at you and (…) saying do this but if she’s (Kate) doing it she can understand (…) the pace.

Pupil 2: She (Kate) doesn’t push you… Like all the teachers tell you to do this, do that, do it as high as possible (…) but she’s like do what you feel is the best for you.

This type of practice demonstrates that by participating in activities with the girls, Kate saw herself as accountable to her pupils as they were to her. Because of this, the girls did not feel as ‘under pressure’ and could work at a level that was best for them. Jess also adopted a similar style when teaching her lessons, as one of her girls commented that she ‘doesn’t just stand there and tell us to do things, she’ll (…) do it with us’. Both teachers participated in physical activities with their classes and, in turn, demonstrated a more democratic practice in comparison to some of their colleagues who ‘stood and shouted’. They were willing to let go of their ‘power authority’ over the girls, and the girls responded positively to this. This suggests that the girls viewed the more traditional practice, that they were used to previously, as one where their teachers were rarely ‘part’ of the lesson and simply just told them what to do, often by shouting. In keeping with the activist pedagogy, Kate and Jess understood that if they joined in with their class (or democratized their practice), this would facilitate the girls’ engagement in their PE lessons.

Another critical element of the activist approach is for teachers to listen and respond to girls’ views in order to co-create PE with girls programs that better meet their needs. Indeed, other activist researchers draw on Cook-Sather’s [28] notion of teachers authorizing their pupils’ voices so that they can begin to see the world from their pupils’ perspectives. This authorization leads to a disruption of the traditional teacher-pupil power relation [11], and thus, to a more democratic practice. This democratic process is a feature of the activist approach through key practices, such as the cyclic process of the Student-Centered Inquiry as Curriculum (SCIC)–see [24].

One girl from School A commented that one task they did was when they ‘listed (…) 6 things on the board and we go around everyone and everyone mentions something’. This process helped the ‘shyer people’ in the class, and the girls noticed that those people now had more say in the ‘choices’ of activities they would do. Another girl commented that this process also helped her ‘(gain) more confidence to (…) speak out in the class’.

Girls from School B also described how their teacher, Kate was able to co-construct their PE experiences that, in turn, led to them being more motivated:

She lets us pick (…) our own things, like not choices but she let us (…) kinda pick metafit… like she asked what do you want to do and we had to write on the paper (…) what we wanted to do (…) [for] activities.

(in PE)

They added that some of the activities they wrote on the paper were fitness, metafit, games, spin class, thighs, bum and tums and that their teacher took all of their suggestions in and ‘(put) it into different lessons’. The activities that were suggested by the girls were observed by the lead researcher as features of their PE program during the activist intervention. This suggests that the decision of what physical activity was being taught was not Kate’s decision, but it was drawn from discussions and writing exercises with the girls. Subsequently, the girls were key drivers of their physical education curricula, and the teacher-pupil power dimension was disrupted.

By co-constructing their PE curricula with the girls, Kate and Jess moved away from the traditional teacher-led approach of ‘I Say, You Do’ and adopted more democratizing practices. This led to pupils feeling more confident to speak out, less pressured during lessons, and more motivated to participate in PE. We know that for pupils to develop lifelong physical activity habits, they must first be willing to engage in activity. The activist approach facilitated the type of environment these girls needed to be willing to participate.

## 4. Discussion

Both studies reveal that pupils responded favorably, both in motivation and emotion, to pedagogies of affect in physical education. The findings from both studies suggest that it is crucial to consider class settings, a sense of connectedness, and social dynamics in class when considering the social environment for enhancing pupils’ motivation, self-determination, and engagement. Notably, the findings offer a basis for developing pedagogies of affect that have the potential to support young people’s mental health and wellbeing.

The issue raised by Study 1 in terms of pupils’ responses to pedagogies of affect was the importance of social support from teachers. A common notion emerged from pupils in girls-only classes, that teachers always encourage pupils, but do not ‘force’ them if pupils are not able or willing to perform a certain task. This point was also shown in Study 2. The girls in Study 2 specifically commented on making more effort when they were not being forced. The data from both studies suggest this teaching behavior makes pupils motivated and determined in the end. This data also gives us an insight into more teacher-directed approaches that continue to persist in physical education [7]. Although we cannot assume teachers forcing pupils to do something is common practice, we can speculate that this type of practice does little to motivate them.

Furthermore, pupils mentioned the importance of connectedness with teachers, which is shown to play a significant role in predicting the levels of pupils’ motivation and engagement in lessons [13]. However, there was a case in Study 1 where a pupil had a negative reaction to the relationship with his teacher. Teachers might need to offer experiences of feeling a sense of connection with others, since this feeling brings promising outcomes, such as learning associated with personal qualities. This suggests teachers’ everyday practice for building trusting relationships with pupils has important implications for the practice of pedagogies of affect.

Study 1 showed that teacher’s clarifying expectations for learning outcomes helped enhance pupils’ motivation and engagement. In particular, boys seemed to prefer to receive immediate corrective feedback and explicit instruction from teachers because it could be helpful to improve their technique and performance. This notion could be interpreted within SDT that some aspects of structure are likely to be closely related to autonomy support, whereas other aspects of structure are sometimes related to control [29]. In this sense, teachers might need to adopt the provision of structure without directiveness and clear expectations if pupil autonomy is a goal.

Study 1 also raised an issue around class settings concerning pupils’ perceptions of need-supportive teaching. The class setting is an important influence on areas of affective learning, such as motivation, engagement, and emotional wellbeing [30]. The results showed that teachers who had a girls-only class engaged in more need-supportive teaching and less controlling teaching than teachers who had a coeducational class and boys-only class. Teachers appeared to interact with girls in a more need-supportive way if the class is set as a girls-only class. In Study 1, female teachers had girls-only classes, while male teachers had boys-only classes. Arguably, this matching might work for adolescents because teachers can develop relationships more easily with pupils of the same sex, though we are aware that this is a highly contentious issue [31]. For example, Stidder [32] showed that teachers experienced difficulties in dealing with issues around the physical support of pupils of the opposite sex. In this sense, teaching in a same-sex class might have the advantage of being autonomy-supportive and less controlling, as a basis of implementing pedagogies of affect.

In Study 2, motivating factors of the pupils’ engagement in physical education were confirmed as choice, variety, and novelty in the activities that were part of their core PE lessons. These factors are not necessarily ground-breaking as they are reported elsewhere in the literature as ways to engage girls (see [22,33]. When study after study [20,24,27]) report that choice, novelty, and variety are features that influence student interest, motivation, and/or learning, it is time that physical education teachers rethink their more traditional pedagogical practices. Indeed, Study 2 demonstrated that having control over these factors was directly related to the girls’ self-determination. This level of control was not found to be from the girls individually, but as a collective with their peers and their teachers. We believe that by co-constructing this environment, pupils were able to change their overall perceptions of physical education, which in turn, led to an increase in their engagement levels. This is consistent with other research on activist approaches to teaching physical activity [20,24], and is the type of practice that challenges the traditional norms that continue to dominate the field. Moreover, this change helps us understand the power dimensions that exist not only between teachers and pupils, but also between pupils and their peers. In Study 2, the activist pedagogy had a direct impact on the change in relationships between girls, and towards the end of the study, they got along with their peers better. This feeling of relatedness to their peer group allowed these girls to experience more emotional satisfaction within their class, and therefore, they had a better experience during their core physical education lessons.

Both studies were able to provide us further insight into traditional approaches that are commonplace in physical education. Study 1 showed that as teachers focus on learning within the affective domain, their teaching behaviors help to establish a connectedness with pupils. Study 2 showed that when teachers used an activist approach and were committed to the democratization of their teaching practice, girls felt more comfortable in their environment, which led to a further connection with their teachers and their peers. Learning in the affective domain and the activist pedagogical model are also connected. They both are markedly different ways to approach teaching physical education that challenges the status quo of more traditional, teacher-directed, approaches. Furthermore, they explicitly focus on helping pupils become more confident, motivated, and self-determined individuals, thus contributing to their wellbeing. Learning through these means is a planned-for outcome, rather than a hoped-for byproduct of teaching for other goals.

## 5. Conclusions

In the context of an emerging crisis in the health and wellbeing of CYP, and in particular, mental health, we have argued in this paper that physical educators’ response must go beyond MVPA-centered programs. Indeed, we believe some teachers in secondary schools in Scotland are already ahead of the curve in this regard. Study 1 showed there are teachers already practicing pedagogies of affect, albeit within programs that tend to remain dominated by traditional practices. Their work is nevertheless consistent with CfE and policy for physical education, given these teachers’ focus on the Personal Qualities aspect of learning. Even though the pedagogy of affect in Study 2 took the form of an activist intervention, again situated within programs dominated by traditional practices, the teachers still were able to have positive influences on their pupils in terms of learning within the affective domain.

We think both studies show that pedagogies of affect, which run against the grain of traditional practices, can be implemented in the real-life situations of schools. It is important to note that fieldwork in both studies was completed prior to 2020. The detrimental effects of the coronavirus pandemic are now increasingly well-understood, particularly in relation to mental health, and the mental health of CYP. We suggest the responses of pupils reported here show the possibilities for physical education to make a positive contribution to health and wellbeing. Pedagogies of affect in physical education can only be more pertinent as we consider the future fallout of this pandemic.

## 6. Limitations and Practical Implications

One limitation of this paper is that it reports data from two studies carried out with different purposes at different times. Study 1 reports data from naturally occurring teaching and learning in secondary schools. Study 2 reports data from an intervention intended to change practice. Nevertheless, we think the focus on pupil responses to these various forms of pedagogy of affect highlight similarities across the data sets. We think these favorable pupil responses suggest that pedagogies of affect may be one potential contribution of physical educators to the emerging mental health crisis among CYP. The existence of pedagogies of affect in Scottish secondary schools suggests that teachers may be ahead of the policy curve and that policymakers should attend to practice on the ground, in schools, where health and wellbeing issues among pupils are experienced daily.

## Data Availability

Data generated by these studies is not available for public access since it remains confidential according to the ethical approval process of the University of Strathclyde and is held on secure and password protected servers.
